# Numerical Simulation on the Acoustic Streaming Driven Mixing in Ultrasonic Plasticizing of Thermoplastic Polymers

**DOI:** 10.3390/polym14061073

**Published:** 2022-03-08

**Authors:** Wangqing Wu, Yang Zou, Guomeng Wei, Bingyan Jiang

**Affiliations:** State Key Laboratory of High Performance Complex Manufacturing, School of Mechanical and Electrical Engineering, Central South University, Changsha 410083, China; csuwwq@csu.edu.cn (W.W.); jal8276478@163.com (Y.Z.); guomengweicsu@163.com (G.W.)

**Keywords:** ultrasonic plasticization, micro injection molding, acoustic streaming, melt mixing, fluorescence intensity

## Abstract

The acoustic melt stream velocity field, total force, and trajectory of fluorescent particles in the plasticizing chamber were analyzed using finite element simulation to investigate the acoustic streaming and mixing characteristics in ultrasonic plasticization micro-injection molding (UPMIM). The fluorescence intensity of ultrasonic plasticized samples containing thermoplastic polymer powders and fluorescent particles was used to determine the correlation between UPMIM process parameters and melt mixing characteristics. The results confirm that the acoustic streaming driven mixing occurs in ultrasonic plasticization and could provide similar shear stirring performance as the screw in traditional extrusion/injection molding. It was found that ultrasonic vibrations can cause several melt vortices to develop in the plasticizing chamber, with the melt rotating around the center of the vortex. With increasing ultrasonic amplitude, the melt stream velocity was shown to increase while retaining the trace, which could be altered by modulating other parameters. The fluorescent particles are subjected to a two-order-of-magnitude stronger Stokes drag force than the acoustic radiation force. The average fluorescence intensity was found to be adversely related to the distance from the sonotrodes’ end surface, and fluorescence particles were more equally distributed at higher parameter levels.

## 1. Introduction

Micro-injection molding has become a key technology for the manufacture of micro- and nano-devices due to its high dimensional accuracy and production efficiency [1]. As an innovative variation of micro-injection molding technology, ultrasonic plasticization micro injection molding (UPMIM) has emerged as a new research hotspot thanks to its advantages of high material utilization [2] and low energy consumption [3,4,5]. A small amount of plastic pellets [6,7] or powder [8,9] is dosed into the plasticizing chamber in UPMIM and plasticized by an ultrasonic sonotrode that vibrates under a sufficient pressure. Interfacial friction [10,11] and volumetric viscous dissipation [12] induced by high-frequency vibration have been reported to be the principal heating mechanisms to plasticize polymers.

In several recent investigations, ultrasonic plasticizing has been shown to improve the dispersion of polymer/montmorillonite [13], polymer/carbon nanotubes [14], polymer/PE99 [15], and other composites systems [16]. Since the surface acoustic wave technique normally uses ultrasonic vibrations to produce a steady laminar flow motion [17,18], we speculate that there may be some kind of shear flow behavior of the melt inside the plasticizing chamber during the UPMIM process. Shear rate is a key design parameter for traditional micro-injection molding equipment that uses a screw plasticizing unit, and is often linked to the plasticizing rate and mixing efficiency [19]. The shear rate and extrusion rate can be adjusted by changing the screw size [20,21], balancing the plasticizing rate with the energy consumption. As a screwless plasticization technology [22,23], the process characteristics of UPMIM differ considerably from traditional micro-injection molding techniques [24], but no relevant research was identified to reveal the shear flow behavior during the UPMIM process. Understanding the melt flow and mixing characteristics during the UPMIM process is critical for equipment design and process parameter optimizations.

In this work, finite element simulation and experimental methods were used to investigate the melt stream flow and mixing characteristics during polymer ultrasonic plasticization and to determine whether ultrasonic vibration induced acoustic streaming could provide the same performance as the screw in traditional extrusion and injection molding. The influence of process parameters on the acoustic pressure, melt stream velocity field, total force, and trajectory of the fluorescent particle were determined by solving the first-order frequency domain acoustic field and second-order time domain flow field using second-order perturbation theory. The mixing characteristics of UPMIM were characterized using the fluorescence intensity of the plasticized samples. The fluorescence intensity distribution was obtained using an optical microscope, and the relationship between the process parameters and the mixing characteristics was established using a single-factor experimental approach.

## 2. Simulation

### 2.1. Mathematical Modeling

#### 2.1.1. Thermodynamic Equations

The independent thermodynamic variables in a continuous flow medium are temperature ***T*** and pressure ***p***. In the first law of thermodynamics, entropy per unit mass ***s*** and density per unit mass ***ρ*** are independent variables. The internal energy per unit mass ***ε*** is calculated using Equation (1).

(1) $$d\varepsilon =Tds-pd\left(\frac{1}{\rho }\right)=Tds+\frac{p}{\rho^{2}}d\rho$$

The relationship between ***dε*** and ***dT*** and ***dp*** can be established by the standard Legendre transformation, as shown in Equation (2).

(2) $$\rho d\varepsilon =\left(c_{p}\rho -\alpha_{p}\rho \right)dT+\left(k_{T}p-\alpha_{p}T\right)dp$$

where ***c_p_*** is the isobaric heat capacity per unit mass; ***α_p_*** is the isobaric thermal expansion coefficient; and ***k_T_*** is the isothermal compression coefficient.

#### 2.1.2. Constitutive Equation

The melt flow characteristic is described using the Carreau generalized non-Newtonian fluid model. The melt viscosity ***μ*** is shear rate dependent, exhibiting Newtonian fluid behavior at low shear rates and power-law fluid behavior at high shear rates. The viscosity model is shown in Equation (3).

(3) $$\mu \left(\dot{\gamma }\right)=\mu_{inf}+\left(\mu_{0}-\mu_{inf}\right){\left(1+{\left(\lambda \dot{\gamma }\right)}^{2}\right)}^{\frac{n-1}{2}}$$

where ***μ*_0_**, ***μ_inf_***, ***λ***, and ***n*** are material coefficients. $\dot{\gamma }$ is the shear rate, 1/s; ***μ*_0_** is the viscosity at zero shear rate, Pa s; ***μ_inf_*** is the viscosity at infinite shear rate, Pa s; ***λ*** is the relaxation time, s; and ***n*** is the power law index.

#### 2.1.3. First Order Thermo-Viscosonic Equation

According to the standard first-order perturbation theory, the field ***g*** can be expressed as ***g* = *g*_0_ + *g*_1_**, where ***g*_0_** is the value of the zero-order state and ***g*_1_** is the acoustic perturbation [25,26]. If the acoustic perturbation ***g*_1_** oscillates at the angular frequency ***ω*** of the acoustic excitation, the field ***g*** can be represented as Equations (4) and (5).

(4) $$g_{1}\left(r,t\right)=g_{1}\left(r\right)e^{-i\omega t}$$

(5) $$\partial_{t}g_{1}=-i\omega g_{1}$$

The first-order thermoviscous acoustic equations for mass, momentum, and energy through frequency domain transformation are shown as Equations (6)–(8).

(6) $$-i\omega \alpha_{p}T_{1}+i\omega k_{T}p_{1}=\nabla v_{1}$$

(7) $$-i\omega \rho_{0}v_{1}=\nabla \left(\tau_{1}-p_{1}1\right)$$

(8) $$-i\omega \rho_{0}c_{p}T_{1}+i\omega \alpha_{p}T_{0}p_{1}=k_{0}^{th}\nabla^{2}T_{1}$$

where **1** is the unit tensor.

#### 2.1.4. Second Order Thermo-Viscosonic Equation

According to the second-order perturbation theory, the field ***g*** can be expressed as ***g* = *g*_0_ + *g*_1_ + *g*_2_**, with ***g*_2_** containing the oscillation term and the time constant term. Assuming second-order field time averaging, the second-order thermal viscous acoustic equations for mass, momentum, and energy are represented as Equations (9)–(11).

(9) $$\nabla [\rho_{0}v_{2}+\langle \rho_{0}v_{1}\rangle ]=0$$

(10) $$\nabla [\tau_{2}-p_{2}1-\rho_{0}\langle v_{1}v_{2}\rangle ]=0$$

(11) $$\nabla [k_{0}^{th}\nabla T_{2}+\langle k_{1}^{th}\nabla T_{1}\rangle +\langle p_{1}\tau_{1}\rangle -\left(1-\alpha_{p}T_{0}\right)\langle p_{1}v_{1}\rangle -\rho_{0}c_{p}\langle T_{1}v_{1}\rangle =0$$

The transition between laminar and turbulent flow is represented by Reynolds number ***Re***, which can be calculated using Equation (12).

(12) $$Re=\rho v_{L}d/\mu$$

where $v_{L}$, ***ρ***, ***μ*** are the flow velocity (m/s), density (kg/m^3^), and viscosity (Pa s) of the melt, respectively. The characteristic length ***d*** (m) of a circular pipe is the diameter. According to the calculation, ***Re*** is substantially lower than the transition value of 2300, indicating that the melt flow state in UPMIM process is laminar, which fits the model setting conditions.

#### 2.1.5. Total Force of Fluorescent Particles

Due to the high viscosity of the polymer melt and the micron size of the fluorescent particles, the gravity effect on the fluorescent particles was ignored. The total force of a single suspended particle can be determined by solving the second-order acoustic fields. The total force is comprised of the acoustic radiation force ***F^rad^*** generated by the acoustic wave on the particle and the Stokes drag force ***F^drag^*** produced by the acoustic streaming flow. When migrating at velocity ***v_p_*** in a fluid with flow velocity ***v_m_***, a spherical particle with radius ***a***, density ***ρ_p_****,* and compression property ***k_p_*** is subjected to the acoustic radiation force ***F^rad^*** and Stokes drag force ***F^drag^***, which may be estimated using Equations (13) and (14).

(13) $$F^{rad}=-\pi a^{3}[\frac{2k_{0}}{3}Re[f_{1}*p_{1}*\nabla p_{1}]-\rho_{0}Re[f_{2}*v*.\nabla v]]$$

(14) $$F^{drag}=6\pi \eta a\left(v_{m}-v_{p}\right)$$

where ***k*_0_** is the melt compressibility and the pre-factors $f_{1}$ and $f_{2}$ are given by

(15) $$f_{1}\left(\tilde{k}\right)=1-\tilde{k},\;\mathrm{with}\;\tilde{k}=\frac{k_{p}}{k_{0}}$$

(16) $$f_{2}\left(\tilde{\rho },\tilde{\delta }\right)=\frac{2\left[1-\Gamma \left(\tilde{\delta }\right)\right]\left(\tilde{\rho }-1\right)}{2\tilde{\rho }+1-3\Gamma \left(\tilde{\delta }\right)},\;\mathrm{with}\;\tilde{\rho }=\frac{\rho_{p}}{\rho_{0}}$$

(17) $$\Gamma \left(\tilde{\delta }\right)=-\frac{3}{2}\left[1+i\left(1+\tilde{\delta }\right)\right]\tilde{\delta },\;\mathrm{with}\;\tilde{\delta }=\frac{\delta }{a}$$

### 2.2. Numerical Modeling

**Figure 1:**
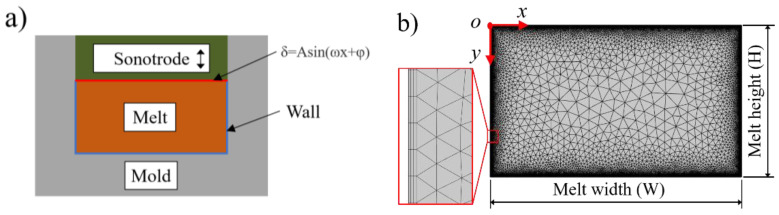
Numerical model: (**a**) boundary conditions; (**b**) finite element mesh.

The numerical modeling was realized in a commercial finite element simulation software COMSOL Multiphysics. Assuming that the polymer is completely plasticized, the computational domain of the numerical model is defined as a rectangular chamber, with a motion boundary on the upper side and to introduce ultrasonic vibration and a wall boundary on the other sides, as shown in Figure 1a. The origin is in the upper left corner of the plasticizing chamber, as illustrated in Figure 1b. The motion boundary is defined as a sinusoidal velocity of δ = Asin (ωx + φ) in the vertical direction, and both the motion boundary and the wall have no-slip conditions. The melt width is the same as the diameter of the ultrasonic sonotrode, which is 10 mm. The melt height varies from 2 to 10 mm with a 2 mm step and is related to the material volume and injection time. The trajectory of the fluorescent particles is realized by the particle tracing for fluid flow interface. Quadrilateral and triangular elements were used to mesh the computational domain. Three-layer quadrilateral elements were used to refine the mesh near the motion boundary and the wall, while the triangular elements were used to mesh the rest zones with a curvature factor of 0.3 and a maximum mesh growth rate of 1.3.

### 2.3. Calculation Scheme

Frequency domain compile equations were used to solve the first-order acoustic field, the stationary compile equations were used to solve the second-order acoustic streaming, and the time dependent compile equations was used to solve particle tracing. A single-factor experimental was used to investigate the acoustic pressure, melt stream velocity field, and total force and trajectory of fluorescent particles in the plasticizing chamber. The ultrasonic amplitude was varied from 20 to 120 μm with a 20 μm increment, while the ultrasonic frequency, the melt height, and the distribution of fluorescent particles were kept constant at 20 kHz, 6 mm, and uniform, respectively. In addition, ultrasonic amplitudes refer to the experimental conditions used in the numerical simulation as well for comparison with the experimental results.

## 3. Experimentation

### 3.1. Material Properties

Polypropylene (Sinopec, PP-T30S, Beijing, China) powder was used in the fluorescence experiment, and its properties are listed in Table 1 [27,28]. Barium magnesium aluminate particles were used as the fluorescent agent and its properties are listed in Table 2.

### 3.2. Methodology

**Figure 2:**
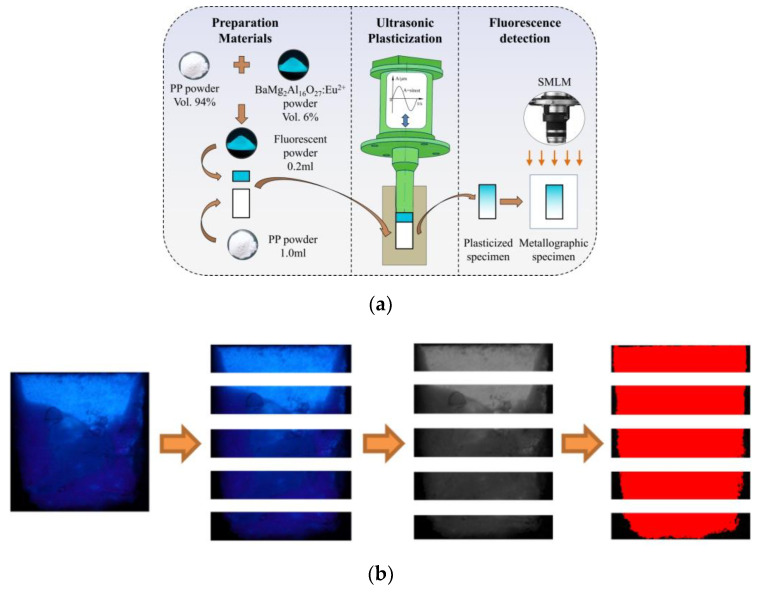
Specimen preparation and characterization. (**a**) Experimental procedures. (**b**) Segmentation and characterization of the fluorescence intensity.

In-house developed UPMIM equipment was used for the ultrasonic plasticization experiments [29]. As shown in Figure 2a, the fluorescent powder was prepared by mixing polypropylene (94% vol) with barium magnesium aluminate (6% vol) for 30 min under oscillating conditions. Next, 1.0 mL of PP powder was placed and compressed in the plasticizing chamber (NAK80 mold steel, 10 mm diameter, and 0–30 mm adjustable height), followed by 0.2 mL of fluorescent powder evenly placed and compressed on top. The ultrasonic vibration energy was subsequently introduced into the plasticizing chamber to fabricate plasticized fluorescent specimens, with an applied ultrasonic amplitude ranging from 56 to 72 μm. The plasticizing pressure, ultrasonic vibration time, holding pressure and holding time were all kept constant at 20 Mpa, 6 s, 20 Mpa, 6 s, respectively. After cutting and grinding, the fluorescence intensity distribution of the fluorescent specimens were analyzed using an optical microscope (VHX-5000, KEYENCE, Osaka, Japan) to investigate the acoustic streaming driven mixing characteristics during ultrasonic plasticization. ImageJ was used to quantify the fluorescence intensity by the gray value of the picture. In more detail, the fluorescence image was cut into pieces in every 2 mm from the top to the bottom. A single channel of the image was extracted and the format was converted to 8-bit grayscale. The threshold function was used to set the grayscale ranging from 5 to 255 to select all fluorescent areas, as illustrated in Figure 2b.

## 4. Results and Discussions

### 4.1. Acoustic Streaming Characteristics

**Figure 3:**
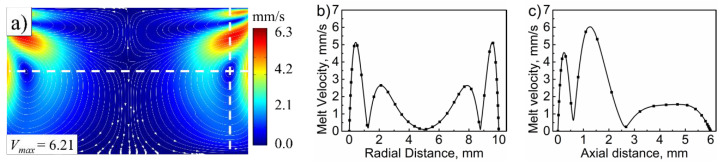
Melt stream velocity distribution in (**a**) the plasticizing chamber, (**b**) radial and (**c**) axial directions along the dash lines (ultrasonic frequency, 20 kHz; ultrasonic amplitude, 80 μm; melt height, 6 mm; fluorescent particles distribution, uniform).

Figure 3 shows the melt stream velocity distribution in the plasticizing chamber under the control parameters. The ultrasonic vibration induced melt disturbance, resulting in the formation of four vortices in the plasticizing chamber (i.e., two small flattened vortices near the end surface of the ultrasonic sonotrode, and two larger and nearly circular vortices near the side wall, all symmetrical along the center axis. Two dashed reference lines were drawn in the center of the larger vortex to quantify the effect of position on the melt stream velocity, as indicated in Figure 3a. The relationship between stream velocity and the position is shown in Figure 3b,c. The melt stream velocity was symmetrical along the central axis. Three minimal stream velocity locations arose in the radial direction: the left main vortex center, the center axis, and the right main vortex center, as shown in Figure 3b. With a maximum flow velocity of 6.21 mm/s, the melt flows around the vortex’s center, resulting in a rather high stream velocity around the vortex’s center, as shown in Figure 3c.

**Figure 4:**
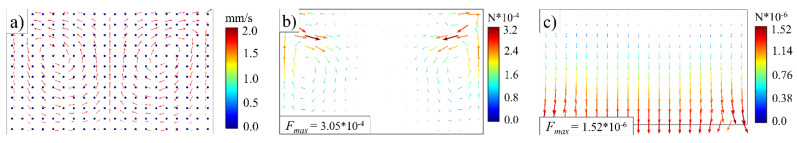
Forces on fluorescent particles (ultrasonic frequency, 20 kHz; ultrasonic amplitude, 80 μm). (**a**) Seeding the fluorescent particles. (**b**) Stokes drag force distribution. (**c**) Acoustic radiation force distribution.

The fluorescent particles of barium magnesium aluminate were seeded uniformly in the model at 0.5 mm intervals to investigate the total force that the acoustic streaming generated during ultrasonic plasticizing, as illustrated in Figure 4a. The total force of the fluorescent particles comprises the acoustic radiation force and the Stokes drag force. The acoustic radiation force can be related to the acoustic pressure difference along the axial direction, and the Stokes drag force is related to the velocity difference between the melt stream and the fluorescent particles. The Stokes drag force distribution under control parameters is comparable to the stream velocity, with a maximum value of 3.05 × 10^−^^4^ N near the vortex, as indicated in Figure 4b. The acoustic radiation force is positively associated with the distance from the top end, with a maximum value of 1.52 × 10^−^^6^ N near the bottom end, as demonstrated in Figure 4c. The Stokes drag force is two orders of magnitude of the acoustic radiation force. Hence, the total force is approximately equal to the Stokes drag force.

**Figure 5:**
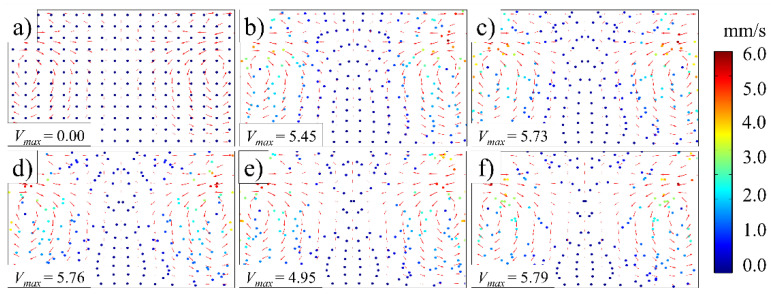
Trajectory of fluorescent particles (ultrasonic frequency, 20 kHz; ultrasonic amplitude, 80 μm): (**a**) 0 s; (**b**) 1 s; (**c**) 2 s; (**d**) 3 s; (**e**) 4 s; (**f**) 5 s.

Figure 5 illustrates the trajectory of fluorescent particles during the ultrasonic plasticizing process. Before the sonotrode vibration, fluorescent particles are evenly distributed in the plasticizing chamber. The fluorescence particles orbit the vortex center as the sonotrode begins to vibrate, and the fluorescence particle can reach a maximum speed of more than 5 mm/s between the two vortices, while it slows down near the center axis and bottom end. The fluorescent particles migrate from the high stream velocity area to the low stream velocity area as the ultrasonic plasticizing time increases, resulting in a steady decrease in Stokes drag force. The velocity of the fluorescent particles decreases as the resistance to motion of the fluorescent particles eventually surpasses the driving force at low stream velocity. The velocity of fluorescent particles is negatively correlated with the current area’s staying time because the number of fluorescent particles tends to be adversely associated with the area velocity. Fluorescent particles accumulated significantly in low stream velocity areas such as the center axis when the ultrasonic plasticizing lasts for 5 s.

### 4.2. Acoustic Streaming Driven Mixing

**Figure 6:**
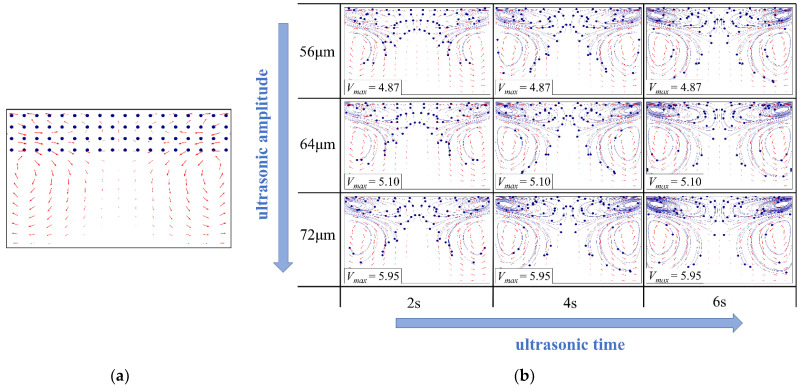
Influence of the ultrasonic amplitude on the trajectory of the fluorescent particles in numerical simulation (ultrasonic frequency, 20 kHz; the ultrasonic amplitudes are all peak-to-peak values of the sinusoidal wave. These are defined according to the allowable range of the ultrasonic vibration system used in the experiment for comparison): (**a**) particle seeding; (**b**) particle tracing.

Fluorescent particles were evenly seeded in an area with a height of 2 mm in the upper section of the plasticizing chamber to investigate the acoustic streaming driven mixing effect, as shown in Figure 6a. The fluorescent particle’s trajectory in numerical simulation at various ultrasonic action time applying the ultrasonic amplitudes of 56, 64, and 72 μm, respectively, as illustrated in Figure 6b. The maximum velocity of the particles increased with increasing ultrasonic amplitude, and the initial acceleration of the particle can be finished in 2 s. The maximum velocity of the particles increased from 4.87 mm/s to 5.95 mm/s when the amplitude increased from 56 μm to 72 μm. The trace of the particle was identical at each ultrasonic amplitude, and all of them rotated around the vortex center. The two small flattened vortices near the end surface of the ultrasonic sonotrode and two larger and nearly circular vortices near the side wall work together to achieve particle mixing. Because the ultrasonic amplitude and action time are proportional to the trace length, particle diffusion rates could be improved by increasing the ultrasonic amplitude and action time.

**Figure 7:**
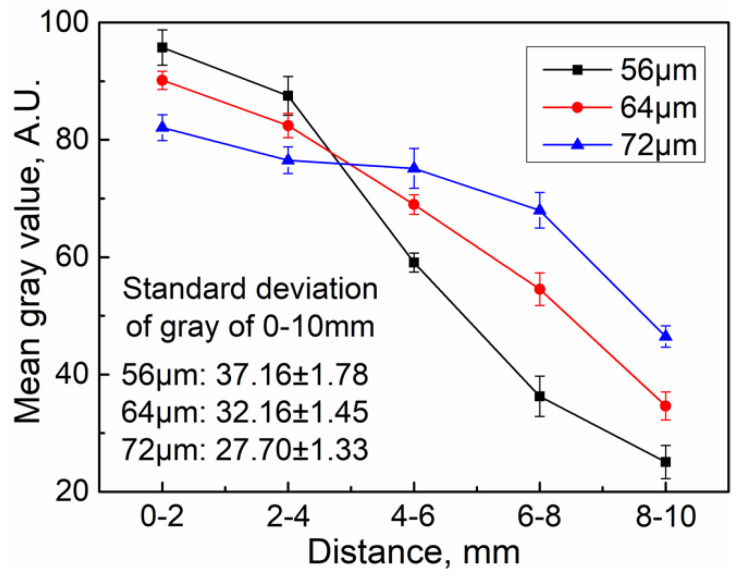
Influence of the ultrasonic amplitude on the mean gray value of the ultrasonic plasticized fluorescent specimens in the experimentation (ultrasonic frequency, 20 kHz; plasticizing pressure, 20 MPa; ultrasonic action time, 6 s; holding pressure, 20 MPa; holding time, 6 s).

Figure 7 shows the influence of the ultrasonic amplitude on the gray value of the ultrasonic plasticized fluorescent specimens in the experimentation. According to our previous investigation [10,11,12], the heat generation rate during ultrasonic plasticizing is significantly influenced by the ultrasonic amplitude. The higher the ultrasonic amplitude, the faster the heat generation rate. Since the heat generation among the particle interfaces lasts only several tens of milliseconds due to the interfacial friction heating, the heat generation with increased ultrasonic amplitude are mainly attributed to the volumetric viscous heating. The difference in the mean gray value of the specimen was the largest when the ultrasonic amplitude was 56 μm. This may be related to the lower heat generation rate during ultrasonic plasticizing, and the melting of the materials is limited, resulting in a poor fluorescent powder diffusion. The difference in the mean gray values became smaller when the amplitude was increased to 72 μm. The polymer heat generation rate is faster at higher amplitudes, leading to an accelerated ultrasonic plasticizing and more melt generation. This further leads to a wider spectrum of fluorescent powder motion along with the melt and a prolonged acoustic streaming driven mixing. Moreover, the improved acoustic streaming driven mixing at increased ultrasonic amplitudes can also be validated by the standard deviation of the mean gray value. The standard deviation of the mean gray value fell from 37.1 to 27.7 when the ultrasonic amplitude was increased from 56 μm to 72 μm. This further verified the numerical simulation results that the fluorescent particles travel faster and further with increased ultrasonic amplitudes, as demonstrated in Figure 6b. Therefore, both the finite element simulation and experimental data illustrate the positive correlation between the diffusion performance of the fluorescent powder and the ultrasonic amplitude. Furthermore, it implies that there is indeed acoustic driven mixing during ultrasonic plasticizing, which could be a comparable but more efficient and energy saving mixing concept than traditional screw-based shear stirring. It should be noted that specimens plasticized at higher amplitudes than 72 μm were not available with the present experimental setup because it may cause cracking of the ultrasonic sonotrode and shift of the resonant frequency.

### 4.3. Analysis of the Influence Mechanism

**Figure 8:**
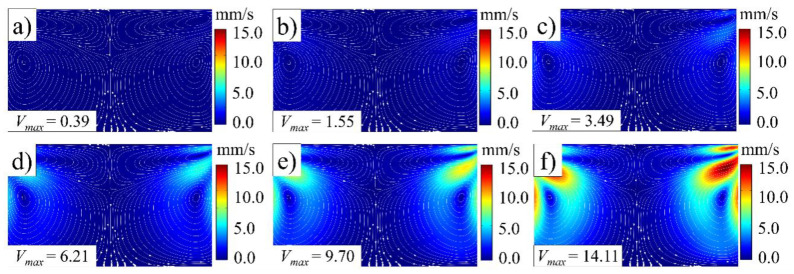
Melt stream velocity distribution under various ultrasonic amplitudes (ultrasonic frequency, 20 kHz): (**a**) 20 μm; (**b**) 40 μm; (**c**) 60 μm; (**d**) 80 μm; (**e**) 100 μm; (**f**) 120 μm.

**Figure 9:**
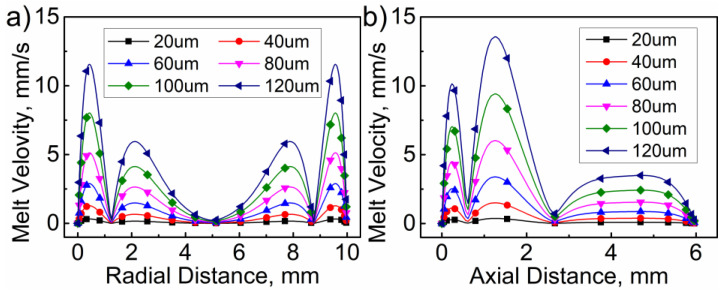
Influence of ultrasonic amplitude on the melt stream velocity in (**a**) radial and (**b**) axial directions.

**Figure 10:**
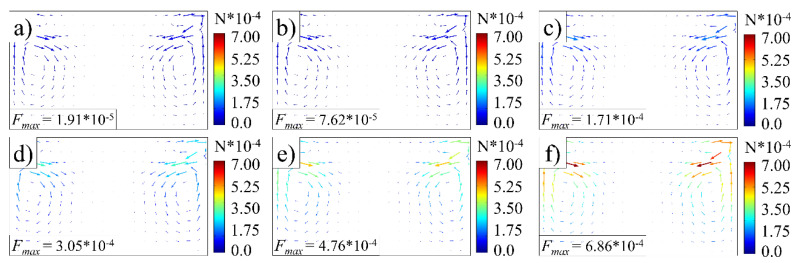
Total force on fluorescent particles at various amplitudes (ultrasonic frequency, 20 kHz): (**a**) 20 μm; (**b**) 40 μm; (**c**) 60 μm; (**d**) 80 μm; (**e**) 100 μm; (**f**) 120 μm.

**Figure 11:**
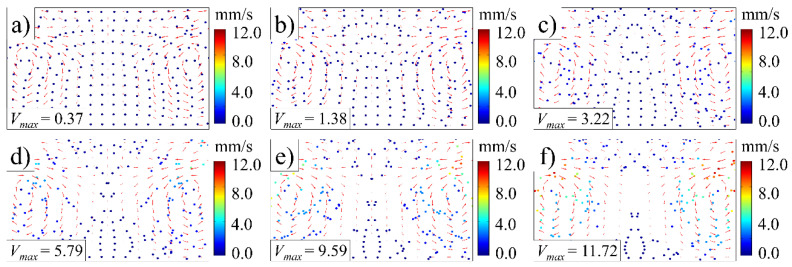
Trajectory of fluorescent particles at various ultrasonic amplitudes (ultrasonic frequency, 20 kHz): (**a**) 20 μm; (**b**) 40 μm; (**c**) 60 μm; (**d**) 80 μm; (**e**) 100 μm; (**f**) 120 μm.

Figure 8, Figure 9, Figure 10 and Figure 11 illustrate the influence of the ultrasonic amplitude on the acoustic melt streaming velocity distribution, the total force on fluorescent particles, and the trajectory of fluorescent particles when the ultrasonic frequency was 20 kHz, respectively. The maximum acoustic streaming velocity increased from 0.39 to 14.11 mm/s, when the ultrasonic amplitude was increased from 20 to 120 μm, as indicated in Figure 8. The ultrasonic amplitude indicated no significant effect on the melt traces, which are mainly affected by the wavelength and shape in the plasticizing chamber. Under varying ultrasonic amplitudes, the melt vortex centers in the plasticizing chamber all appeared at the same locations. With increasing ultrasonic amplitude, the momentum of the plasticized melt increased, as did the acoustic streaming velocity. Hence, the acoustic melt streaming velocity can be increased with increasing ultrasonic amplitude, as illustrated in Figure 9, where a series line graph was acquired by drawing a reference line via the center of the primary vortex in both radial and axial directions.

As discussed in Section 4.1, the total force is approximately equal to the Stokes drag force, which is linearly related to the velocity difference between the melt and the fluorescent particles, according to Equation (14). The melt stream velocity can be increased exponentially with increasing ultrasonic amplitude, as demonstrated in Figure 8. Therefore, it can be drawn that the total force can also be changed exponentially with increasing ultrasonic amplitude. This is indeed the case, as we found in the numerical simulation. The maximum value of the total force increased from 1.91 × 10^−5^ N to 6.86 × 10^−5^ N while maintaining the distribution pattern when the ultrasonic amplitude increased from 20 μm to 120 μm, as illustrated in Figure 10.

Another factor that can be related to the total force distribution of the fluorescent particles is viscous heating induced by ultrasonic excitation. As the ultrasonic vibration energy increases with increasing amplitude, the viscosity of the viscoelastic melt blend decreases due to the energy dissipation and corresponding viscous heating. Normally, the decrement in viscosity can reduce the Stokes drag force according to Equation (14). However, both the acoustic driven vortices and the reduced viscosity could facilitate the melt stream flow. That is, the acoustic driven vortex flow has more significant influence on the Stokes force of the fluorescent particles than that of the viscous heating induced viscosity reduction. Therefore, the total force of the fluorescent particles increases exponentially with increasing amplitudes.

The trajectory of fluorescent particles under various ultrasonic amplitudes is shown in Figure 11. Under lower ultrasonic amplitudes, the fluorescent particles move at slower speeds and travel shorter distances along the traces. The influence of ultrasonic amplitude on the trace is negligible, and it appears to be approximately squared with the fluorescent particle motion velocity. When compared to Figure 6, it is clear that raising the ultrasonic time and amplitude improves the fluorescent particle travel distance while maintaining the traces, leading to an enhanced mixing performance.

## 5. Conclusions

Shear flow behavior during the plasticization process has been the research focus of the design of an injection molding machine. The lack of relevant research has restricted the development of ultrasonic plasticization microinjection molding (UPMIM). In this work, numerical simulations were conducted in COMSOL Multiphysics to investigate the polymer melt stream flow and mixing characteristics in UPMIM. The acoustic pressure distribution, melt stream velocity field, total force, and trajectory of fluorescent particles in the plasticizing chamber were analyzed to uncover the acoustic driven mixing mechanism and possible shear stirring effect in UPMIM. The fluorescence intensity of ultrasonic plasticized samples containing thermoplastic polymer powders and fluorescent particles was used to determine the correlation between UPMIM process parameters and melt mixing characteristics. It was determined that the acoustic streaming driven mixing effect does occur in ultrasonic plasticizing, and could provide similar shear stirring performance as the screw in traditional extrusion/injection molding. Several melt vortices in the plasticizing chamber can be formed due to the ultrasonic vibration. With increasing ultrasonic amplitude, the melt stream velocity was shown to increase while retaining the trace, which could be altered by modulating other parameters. The fluorescent particles are subjected to a two-order-of-magnitude stronger Stokes drag force than the acoustic radiation force. The average fluorescence intensity was found to be adversely related to the distance from the end surface of the ultrasonic sonotrode, and fluorescence particles were more equally distributed at higher parameter levels.

## Figures and Tables

**Table 1:** Material properties of polypropylene.

Density	Acoustic Velocity	Melt Point	* μ_0_	** μ_inf_	Power Index	Particle Size
0.9 g/cm^3^	1623 m/s	170 °C	2000 pa s [27]	10 pa s [28]	0.38	200 mesh

* μ_0_ is the viscosity at zero shear rate. ** μ_inf_ is the viscosity at infinite shear rate.

**Table 2:** Material properties of barium magnesium aluminate.

Formula	Density	Particle Size	Excitation Peaks	Emission Peak
BaMg_2_Al_16_O27:Eu^2+^	5.1 g/cm^3^	200 mesh	395 nm	450 nm

## Data Availability

The raw/processed data required to reproduce these findings cannot be sheared at this time due to technical or time limitations.

## References

[1] Qin Y., Brockett A., Ma Y., Razali A., Zhao J., Harrison C., Pan W., Dai X., Loziak D. (2010). Micro-manufacturing: Research, technology outcomes and development issues. Int. J. Adv. Manuf. Technol..

[2] Sacristán M., Plantá X., Morell M., Puiggalí J. (2014). Effects of ultrasonic vibration on the micro-molding processing of polylactide. Ultrason. Sonochem..

[3] Heredia-Rivera U., Ferrer I., Vázquez E. (2019). Ultrasonic Molding Technology: Recent Advances and Potential Applications in the Medical Industry. Polymers.

[4] Jiang B., Zou Y., Wei G., Wu W. (2019). Evolution of Interfacial Friction Angle and Contact Area of Polymer Pellets during the Initial Stage of Ultrasonic Plasticization. Polymers.

[5] Dorf T., Ferrer I., Ciurana J. (2018). Characterizing Ultrasonic Micro-Molding Process of Polyetheretherketone (PEEK). Int. Polym. Process..

[6] Dorf T., Perkowska K., Janiszewska M., Ferrer I., Ciurana J. (2018). Effect of the main process parameters on the mechanical strength of polyphenylsulfone (PPSU) in ultrasonic micro-moulding process. Ultrason. Sonochem..

[7] Grabalosa J., Ferrer I., Elías-Zúñiga A., Ciurana J. (2016). Influence of processing conditions on manufacturing polyamide parts by ultrasonic molding. Mater. Des..

[8] Sánchez-Sánchez X., Hernández-Avila M., Elizalde L.E., Martínez O., Ferrer I., Elías-Zuñiga A. (2017). Micro injection molding processing of UHMWPE using ultrasonic vibration energy. Mater. Des..

[9] Ferrer I., Vives-Mestres M., Manresa A., Garcia-Romeu M.L. (2018). Replicability of Ultrasonic Molding for Processing Thin-Wall Polystyrene Plates with a Microchannel. Mater. Des..

[10] Wu W., Peng H., Jia Y., Jiang B. (2017). Characteristics and mechanisms of polymer interfacial friction heating in ultrasonic plasticization for micro injection molding. Microsyst. Technol..

[11] Peng T., Jiang B., Zou Y. (2019). Study on the Mechanism of Interfacial Friction Heating in Polymer Ultrasonic Plasticization Injection Molding Process. Polymers.

[12] Jiang B., Peng H., Wu W., Jia Y., Zhang Y. (2016). Numerical Simulation and Experimental Investigation of the Viscoelastic Heating Mechanism in Ultrasonic Plasticizing of Amorphous Polymers for Micro Injection Molding. Polymers.

[13] Planellas M., Sacristán M., Rey L., Olmo C., Aymamí J., Casas M.T., del Valle L.J., Franco L., Puiggalí J. (2014). Micro-molding with ultrasonic vibration energy: New method to disperse nanoclays in polymer matrices. Ultrason. Sonochem..

[14] Olmo C., Amestoy H., Casas M.T., Martínez J.C., Franco L., Sarasua J.-R., Puiggalí J. (2017). Preparation of Nanocomposites of Poly (ε-caprolactone) and Multi-Walled Carbon Nanotubes by Ultrasound Micro-Molding. Influence of Nanotubes on Melting and Crystallization. Polymers.

[15] Diaz A., Franco L., Casas M.T., Del Valle L.J., Aymamí J., Olmo C., Puiggalí J. (2014). Preparation of micro-molded exfoliated clay nanocomposites by means of ultrasonic technology. J. Polym. Res..

[16] Michaeli W., Kamps T., Hopmann C. (2011). Manufacturing of polymer micro parts by ultrasonic plasticization and direct injection. Microsyst. Technol..

[17] Destgeer G., Lee K.H., Jung J.H., Alazzam A., Sung H.J. (2013). Continuous separation of particles in a PDMS microfluidic channel via travelling surface acoustic waves (TSAW). Lab Chip.

[18] Shi J., Huang H., Stratton Z., Huang Y., Huang T.J. (2009). Continuous particle separation in a microfluidic channel via standing surface acoustic waves (SSAW). Lab Chip.

[19] Boronat T., Segui V., Peydro M., Reig M.J. (2009). Influence of temperature and shear rate on the rheology and processability of reprocessed ABS in injection molding process. J. Mater. Process. Technol..

[20] Wyman C.E. (1975). Theoretical model for intermeshing twin screw extruders: Axial velocity profile for shallow channels. Polym. Eng. Sci..

[21] Michaeli W., Opfermann D. (2006). Ultrasonic plasticising for micro injection moulding. Proceedings of the 4M 2006—Second International Conference on Multi-Material Micro Manufacture.

[22] Wilczyński K., Nastaj A., Lewandowski A., Wilczyński K.J., Buziak K. (2019). Fundamentals of Global Modeling for Polymer Extrusion. Polymers.

[23] Yu H.W., Lee C.H., Jung P.G., Shin B.S., Kim J.-H., Kwang K.-Y., Ko J.S. (2009). Polymer microreplication using ultrasonic vibration energy. J. Micro/Nanolithogr. MEMS MOEMS.

[24] Zou Y., Wu W., Zhou X., Wei G., Jiang B. (2021). A novel method for the quantitative characterization of the simultaneous plasticizing and filling performance in ultrasonic plasticization micro injection molding. Mater. Des..

[25] Muller P.B., Barnkob R., Jensen M.J.H., Bruus H. (2012). A numerical study of microparticle acoustophoresis driven by acoustic radiation forces and streaming-induced drag forces. Lab Chip.

[26] Karlsen J.T., Bruus H. (2015). Forces acting on a small particle in an acoustical field in a thermoviscous fluid. Phys. Rev. E.

[27] Martienssen W., Warlimont H. (2006). Springer Handbook of Condensed matter And Materials Data.

[28] Chun K.S., Husseinsyah S., Yeng C.M. (2015). Torque rheological properties of polypropylene/cocoa pod husk composites. J. Thermoplast. Compos. Mater..

[29] Jiang B., Zou Y., Liu T., Wu W. (2019). Characterization of the Fluidity of the Ultrasonic Plasticized Polymer Melt by Spiral Flow Testing under Micro-Scale. Polymers.

